# Comparison of the efficacy and safety of acupuncture and acupotomy for patients with cervical spondylotic radiculopathy: A protocol for systematic review and meta-analysis

**DOI:** 10.1097/MD.0000000000025239

**Published:** 2021-04-02

**Authors:** Shiming Zhou, Biao Liu, Jinwei Guo

**Affiliations:** Department of Orthopedics, Jiangjin District Central Hospital, Chongqing, China.

**Keywords:** acupotomy, acupuncture, cervical spondylotic radiculopathy, meta-analysis, protocol, systematic review

## Abstract

**Background:**

There is no systematic review to compare the efficacy of acupuncture and acupotomy in patients with cervical spondylotic radiculopathy. It is worthy to critically review the evidence of the comparison of these 2 therapies to inform clinical practice. Therefore, the purpose of this study was to compare the efficacy and safety of acupuncture and acupotomy in the treatment of cervical spondylotic radiculopathy and to provide evidence for clinical practice.

**Methods:**

Seven electronic databases including Web of Science, Embase, PubMed, Wanfang Data, Scopus, Science Direct, Cochrane Library were searched in March 2021 by 2 independent reviewers. Data extraction was performed independently, and any conflict was resolved before final analysis. Only randomized clinical trials were included in this study. Outcomes included pain intensity, symptom score, neck disability index, total effective rate, and curative rate. The Cochrane risk of bias tool is used to evaluate the risk of bias of included randomized controlled trials by 2 independent reviewers.

**Results:**

We hypothesized that these 2 methods would provide similar therapeutic benefits. The results of this research will be delivered in a peer-reviewed journal.

**Conclusion:**

This study expects to provide credible and scientific clinical evidence for the efficacy and safety of acupuncture and acupotomy in the treatment of cervical spondylotic radiculopathy.

**OSF registration number:**

10.17605/OSF.IO/U7T6A.

## 1 Introduction

Cervical spondylotic radiculopathy is characterized by the dysfunction of a cervical spinal nerve, the roots of the nerve, or both.^[1,2]^ Patients with cervical spondylotic radiculopathy suffer deeply from numbness and pain of the arm and neck. According to the Global Burden of Disease Study (2013),^[3]^ among 301 chronic and acute injuries and illnesses in 188 countries, neck pain was one of the top 10 causes of disability of years. Furthermore, neck activities are restricted. Cervical spondylotic radiculopathy, as a common type of cervical spondylosis, accounts for about 60% to 70%.^[4]^

Management of cervical spondylotic radiculopathy includes surgical and conservative treatment. Typically, patients should be treated conservatively for at least 6 weeks, and surgical intervention should be considered in patients with no improvement after 6 to 12 weeks of nonoperative treatment, significant muscle weakness, progressive neurological deficits, or myelopathy.^[5,6]^ Nonoperative treatment is the first choice for most patients because of surgery-related adverse events and recurrence. Non-surgical treatment improves symptoms in 75% to 90% of patients. Currently, non-surgical treatment includes anti-inflammatory drug therapy, physical therapy, epidural steroid injection procedures, cervical spine traction, exercise, acupotomy, massage, and various combinations of these.^[7,8]^

Acupotomy, also named needle knife, which resembles both Chinese acupuncture therapy and modern surgical principles, is a Traditional Chinese Medicine intervention widely used to treat neck pain and other diseases. Some hypotheses has been suggested that needle knife is to release the diseased tissue, relieve the stimulation or compression of nerves and blood vessels, and restore the ameliorate pain and numbness symptoms. Other studies have suggested that needle knife induces natural opioid-mediated pain suppression by stimulating local a-delta nerve fibers.^[9–11]^

Acupotomy has the characteristics of both acupuncture and microinvasive operation. Acupuncture and acupotomy have been widely used in China to treat cervical spondylotic radiculopathy with satisfactory results. However, there is no systematic review or meta-analysis to compare the efficacy of these 2 therapies in patients with cervical spondylotic radiculopathy. It is worthy to critically review the evidence of the comparison of these 2 therapies to inform clinical practice. Therefore, the purpose of this study was to compare the efficacy and safety of acupuncture and acupotomy in the treatment of cervical spondylotic radiculopathy and to provide evidence for clinical practice. We hypothesized that these 2 methods would provide similar therapeutic benefits.

## 2 Materials and methods

### 2.1 Protocol registration

The prospective registration has been approved by the open science framework (OSF) registries (https://osf.io/b3yxd), and the registration number is 10.17605/OSF.IO/B3YXD. The protocol was written following the preferred reporting items for systematic reviews and meta-analyses protocols (PRISMA-P) statement guidelines.

### 2.2 Selection of studies

**Figure 1:**
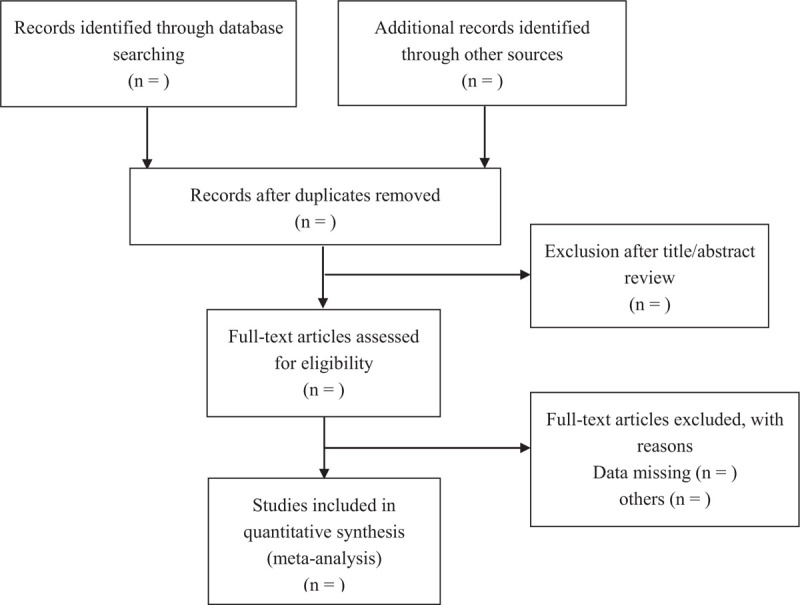
PRISMA flow diagram describing the selection process for relevant clinical trials used in this meta-analysis. PRISMA = Preferred Reporting Items for Systematic Reviews and Meta-Analyses.

Seven electronic databases including Web of Science, Embase, PubMed, Wanfang Data, Scopus, Science Direct, Cochrane Library were searched in March 2021 by 2 independent reviewers. The established search strategy for PubMed was displayed in Table 1. The reference lists of the included studies were also checked for additional studies that were not identified with the database search. There was no restriction in the dates of publication or language in the search. No ethical approval was required in our study because all analyses were based on aggregate data from previously published studies (Fig. 1).

**Table 1:** Search strategy of PubMed.

Number	Search items
Mesh term #1	((acupotomy) OR (acupotome) OR (needle knife) OR (needle scalpel) OR (acupotomlogy) OR (miniscalpel acupuncture) OR (miniscalpel needle) OR (stiletto needle) OR (sword like needle) OR (Xiaozhendao))
Mesh term #2	((acupuncture) OR (manual acupuncture) OR (auricular acupuncture) OR (scalp acupuncture) OR (fire needling) OR (warm needling) OR (electro-acupuncture))
Mesh term #3	((cervical radiculopathy) OR (cervical spondylotic radiculopathy) OR (cervical spondylopathy) OR (cervical spondylosis) OR (neck pain) OR (neck syndrome)
Mesh term #4	((clinical trials) OR (random control trials))
#1 and #2 and #3 and #4	

### 2.3 Inclusion and exclusion criteria

Study included in this systematic review and meta-analysis had to meet all of the following inclusion criteria in the PICOS order: participants: patients with cervical spondylotic radiculopathy; intervention: patients received acupuncture; comparator: patients received acupotomy; outcomes: outcomes which assessed pain intensity, symptom score, neck disability index, total effective rate, and curative rate; study design: randomized controlled trials. The exclusion criteria were as follows: studies which did not assessed the above outcomes; no direct comparison of acupuncture and acupotomy; studies with the following types: case reports, comments or letters, biochemical trials, protocols, conference abstracts, reviews, and retrospective studies or prospective non-randomized studies.

### 2.4 Study selection

Articles were exported to EndNote, and duplicates removed. Two independent authors screened the titles and abstracts of potentially relevant studies to determine their eligibility based on the criteria. Disagreements were resolved through a discussion with a third review author.

### 2.5 Data extraction

Data were extracted by review of each study for population, mean age, sex, follow-up duration, study design, publishing date, acupuncture and acupotomy characteristics, and outcomes assessment. The 2 reviewers created a study-specific speadsheet in Excel (Microsoft Corp. Washington) for data collection. Data extraction was performed independently, and any conflict was resolved before final analysis. Any disagreements between the 2 reviewers were discussed and, if necessary, the third author was referred to for arbitration. If the data were missing or could not be extracted directly, authors were contacted by email. Otherwise, we calculated them with the guideline of Cochrane Handbook for Systematic Reviews of Interventions 5.1.0. If necessary, we would abandon the extraction of incomplete data.

### 2.6 Quality assessment

The GRADE system (Grading of Recommendations Assessment, Development and Evaluation) was used by 2 independent reviewers to rate the overall quality of evidence in each pooled analysis. The following 7 items were used to assess the quality of randomized controlled trials: random sequence generation, allocation concealment, blinding of participants and personnel, blinding of outcome assessment, incomplete outcome data, selective reporting, and other bias. The quality rating high is reserved for evidence based on randomized controlled trials. The quality rating moderate, low, or very low were rated depending on the following 4 factors: risk of bias, inconsistency of effect, imprecision, and indirectness. When the heterogeneity was high, inconsistency was considered serious. When there was no direct comparison between acupuncture and acupotomy, indirectness was considered serious and researchers had to make comparisons across studies. When there was fewer than 400 participants for each outcome, imprecision was considered an appreciable risk. Any controversy was resolved by discussing with a third author to reach a final consensus.

### 2.7 Statistical analysis

Data analysis was performed with Review Manager Software (RevMan Version 5.4, The Cochrane Collaboration, Copenhagen, Denmark). As outcomes which assessed pain intensity might be reported on different scores, we used the standardized mean difference with a 95% confidence interval to assess for these outcomes. A *P* value <.05 was considered statistically significant. All outcomes were pooled on random-effect model. The statistical heterogeneity was assessed by using the Cochrane Q test and *I*^2^ statistic. The low, moderate, and high heterogeneity were assigned to *I*^2^ values of 0% to 25%, 26% to 74%, and above 75%. A meta-analysis was conducted when ≥4 trials reported an outcome of interest. A sensitivity analysis was planned by different follow-up periods. Begg funnel plot was used to assess publication bias. If publication bias exists, the Begg funnel plot is asymmetric.

## 3 Discussion

To the best of our knowledge, this is the first study to date to compare the efficacy and safety of acupuncture and acupotomy in the treatment of cervical spondylotic radiculopathy. We hypothesized that these 2 methods would provide similar therapeutic benefits. We conducted this systematic review and meta-analysis according to the PRISMA guidelines. Two independent authors used a highly sensitive search strategy to identify the trials in the 7 main databases and supplemented it by manually searching for studies related to the topic and the reference list of included studies. There was no restriction in the dates of publication or language in the search for the current review, and thus publication and language bias could be minimized. However, the studies that were only indexed in the local database might be missed and therefore were not included in our study. In accordance with recommendations of GRADE, the quality of the evidence was carefully evaluated in this review, and thus generating a precise level of confidence of our results.

## Author contributions

**Data curation:** Shiming Zhou.

**Formal analysis:** Shiming Zhou, Biao Liu.

**Funding acquisition:** Jinwei Guo.

**Investigation:** Shiming Zhou, Biao Liu.

**Methodology:** Shiming Zhou, Biao Liu.

**Project administration:** Jinwei Guo.

**Resources:** Jinwei Guo.

**Software:** Biao Liu.

**Supervision:** Jinwei Guo.

**Validation:** Shiming Zhou, Jinwei Guo.

**Writing – original draft:** Shiming Zhou, Biao Liu.

**Writing – review & editing:** Jinwei Guo.
